# Immune surveillance and microbial escape in the aging host: Why does the microbiome lose its balance?

**DOI:** 10.1371/journal.pbio.3003815

**Published:** 2026-05-20

**Authors:** Siqi Liu, Flávio Silva Costa, Dario Riccardo Valenzano

**Affiliations:** 1 Leibniz Institute on Aging, Fritz Lipmann Institute (FLI), Jena, Germany; 2 Friedrich Schiller University, Jena, Germany; 3 Cluster of Excellence Balance of the Microverse, Friedrich Schiller University, Jena, Germany

## Abstract

Host-associated microbiomes are compositionally stable across most of the life span, yet undergo consistent and marked deterioration during aging, a phenomenon linked to metabolic dysfunction and disease. What drives this late-life collapse remains poorly understood, in part because the mechanisms by which hosts actively construct and maintain the microbial niche during adulthood remain incompletely characterized. This Unsolved Mystery integrates evidence from immunology and ecosystem ecology to investigate the role of immunosenescence in age-associated dysbiosis, raising the possibility of interventions that restore immune surveillance capacity alongside ecologically informed microbiome management, rather than targeting community composition in isolation.

## Abstract 2

Host-associated microbiomes are stable across most of the lifespan, but what drives late-life deterioration remains unclear. This Unsolved Mystery proposes interventions that restore immune surveillance capacity alongside ecologically informed microbiome management.

## Introduction

To understand the human host–microbiome relationship and how it changes with age, it is useful to begin from a broader perspective. Multicellular life evolved in an environment populated by single-cell organisms capable of complex biochemical reactions, optimized over several billion years of unicellular life. The evolution of multicellular life led to privileged partnerships between multicellular ‘hosts’ and subsets of the surrounding microbes, including archaea, bacteria, and single-celled eukaryotes [1,2]. Progressive ecological niche specialization in multicellular organisms led to the establishment of host-specific microbial consortia, which participate in virtually all aspects of host biology [3]. Co-evolution of multicellular hosts and their associated microbial communities resulted in a range of interactions, from loose opportunistic associations, to extreme cases of co-phylogeny, where specific microbial lineages diversified in strict dependence with the host lineage [4,5]. While phylogeny shaped species-specific microbiota community structure and function, during ontogeny, individual trajectories of microbial communities become specific to individual hosts, reflecting and impacting an individual’s health, diet, and lifestyle, as well as genetic makeup [6].

Host-associated microbial consortia are not passive bystanders but active participants in virtually all aspects of host biology. They contribute essential metabolic functions (including synthesis of vitamins, bile acid transformation, and production of short-chain fatty acids), support immune system maturation [7], provide colonization resistance against pathogens, and mediate nutrient absorption [3]. The depth of these dependencies is illustrated by the diversity of symbiotic arrangements they have produced: from the bobtail squid’s luminescent partnership with *Aliivibrio fischeri* [8], to the obligate dependence of termites on hindgut flagellates and their associated archaea for cellulose digestion [9], to the selective enrichment of *Bifidobacterium* in the human infant gut through prebiotic human milk oligosaccharides [10]. Beyond bacteria, host-associated communities spanning archaea and single-celled eukaryotes are now recognized as functionally integrated components of host metabolism, the contributions of which have been systematically underestimated by bacteria-centric sequencing approaches [11].

Germ-free organisms have provided an invaluable experimental window into the causal contributions of individual microbial actors to host physiology, development, and immunity. Yet, they can only be maintained under highly stringent, artificially controlled conditions, a practical reminder that for most multicellular life, the absence of a microbiome is not a natural state but an experimentally imposed one [12]. Experimental evidence from multiple model systems demonstrates that deliberate microbial manipulations are sufficient to alter aging trajectories and life span, underscoring the causal rather than merely correlative nature of the microbiome–aging relationship [13–15]. What, then, sustains microbiome stability across the life span, and why does this stability fail with age?

Although immune–microbiome interactions have been already documented, in this Unsolved Mystery, we propose that immune surveillance operates as a central organizing principle of microbial ecology throughout life, continuously constraining microbial proliferation and suppressing community compositional drift. Integrating evidence from immunology and ecosystem ecology, we argue that immunosenescence progressively dismantles this control, enabling competitive escape dynamics analogous to those observed in kill-the-winner phage–bacteria systems and tumor immunoevasion. This framework reframes age-associated dysbiosis not as passive drift but as a failure of active host-mediated control. It points toward interventions that restore immune surveillance capacity alongside ecologically informed microbiome management, rather than targeting community composition in isolation.

## How do microbiomes assemble in early life?

The first encounters between a naive host and its future microbial partners are not entirely left to chance. In organisms born largely microbe-free, initial colonization can occur passively through environmental exposure. In zebrafish, for instance, gut symbionts are recruited horizontally from surrounding water and food [16]. In humans, the mode of delivery shapes initial colonization decisively. Vaginally delivered infants acquire a microbiome resembling maternal vaginal and intestinal communities, dominated early by *Lactobacillus* and other facultative anaerobes that prepare the gut environment for subsequent colonization, whereas C-section-delivered infants acquire a community resembling maternal skin and the hospital environment, with delayed and altered acquisition of Bacteroides species and persistent compositional differences that are detectable for months after birth [17]. Whether these differences leave durable physiological signatures into adulthood remains debated, but the case illustrates how a single perinatal event can shift the trajectory of an otherwise stochastic assembly process. Yet across distantly related taxa, a more active logic operates in parallel: parents actively manage the microbial landscape to which their offspring will be exposed. Burying beetles of the genus *Nicrophorus* chemically remodel the carcass on which their larvae will feed, selectively suppressing competing decomposers while inoculating the substrate with a core symbiotic community transmitted via oral and anal secretions [18,19]. Discus fish achieve a functionally analogous outcome through a different route, with both parents feeding hatchlings directly from their skin mucus for the first weeks of life [20]. In humans, the production of milk oligosaccharides (complex sugars indigestible by the infant yet selectively metabolized by *Bifidobacterium*) represents a biochemically sophisticated version of the same principle: a parent actively managing the nutritional landscape to favor a specific microbial outcome in the offspring’s gut [21].

The transition from milk to solid food at weaning marks a second major succession event in the human gut microbiome. Longitudinal cohort data identify three distinct developmental phases, with cessation of breastfeeding emerging as the single most important driver of microbiome maturation towards an adult-like Firmicutes-dominated consortium, in a transition that takes one to three years to complete [17]. Perinatal colonization and weaning together establish the adult community. The maintenance of this community is the central concern of this Unsolved Mystery. That such mechanisms have evolved independently across insects, fish, and mammals reveals a continuum of increasing parental investment in offspring microbiome assembly: from passive exposure, to active chemical curation of the surrounding microbial landscape, to vertical transmission itself. At this extreme, the host bypasses environmental contingency entirely, directly seeding the offspring with its own microbial community. Tortoise leaf beetles illustrate how far this logic can be taken: females provision each egg with a symbiont-laden caplet produced in dedicated ovary-associated glands, which the larva consumes during embryonic development, before it has encountered a single environmental microbe [22]. However, for most hosts, the challenge is not securing a single obligate partner, but assembling and sustaining a complex, multi-kingdom microbial community in a process that unfolds over developmental time and is shaped by both internal and environmental forces.

The degree to which host-associated microbial communities are stable throughout life varies considerably across taxa, reflecting differences in host longevity, developmental strategy, and the ecological contexts in which host–microbiome partnerships evolved. In species where community dynamics have been examined longitudinally, most extensively in humans and other mammals, a recognizable pattern emerges: after an early period of assembly and succession, microbial communities reach a compositional equilibrium that is maintained with surprising fidelity across adulthood, before deteriorating in old age. Microbiome assembly reflects the interplay of deterministic and stochastic forces operating at different scales. Early colonization is contingent on the founding taxa, arrival sequence, and the ambient microbial environment at birth, all of which introduce variation with lasting compositional consequences. Yet communities across individuals repeatedly converge on similar functional architectures, a pattern consistent with host-dependent selection, shared environmental filtering, or intrinsic ecological assembly rules, and likely reflecting all three. Stochasticity and determinism are not competing explanations but complementary ones, each dominant at a different phase of the host–microbiome relationship. This pattern of stochastic origins progressively giving way to convergent community order, to which the host actively contributes through niche construction, frames the host–microbiome relationship as one of ongoing host engagement rather than of passive cohabitation [23,24]: the host does not merely filter among available microbes but adds an active, self-reinforcing layer of immunological and physicochemical constraint on top of ecological and environmental forces. What mechanisms sustain this constructed niche across decades of adult life, and what happens when they fail, are the central questions addressed in this Unsolved Mystery.

## Immune surveillance as an organizing principle of microbiome homeostasis

**Fig 1:**
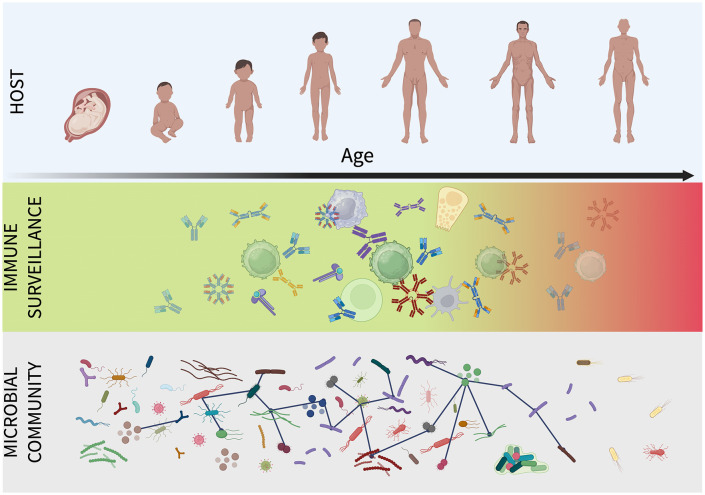
Immune surveillance maintains microbiome homeostasis across the life span. Top: Host developmental trajectory from prenatal life through to old age. Middle: Mucosal immune populations central to microbial surveillance, including IgA-producing plasma cells, naive T cells, mucosal-associated invariant T cells (MAIT cells), innate lymphoid cells (e.g., ILC3s), and innate effectors. The green-to-red gradient depicts dual-axis remodeling with age: progressive erosion of adaptive precision (declining naive lymphocyte output, contracting receptor diversity, reduced mucosal IgA specificity) accompanied by a rise in constitutive, poorly discriminating inflammatory tone. Bottom: The host-associated microbial community, represented as taxa connected by ecological interaction networks. In adulthood, intact surveillance sustains a diverse, evenly distributed, interconnected community. In aging, the combination of eroded specificity and a pro-inflammatory background enables expansion of opportunistic taxa, loss of core commensals, and breakdown of community network structure. Figure created in BioRender. *Valenzano, D. R. (2026)* *https://BioRender.com/q1bi52p*.

Niche construction describes the outcome, specifically a host-engineered environment that progressively filters and constrains its microbial community, but it does not name the mechanism. The microbiome can be thought of as an “ecosystem on a leash” [25], actively held in check by the host. What biological force, operating continuously across decades of adult life, actively suppresses microbial proliferation, distinguishes tolerable from intolerable community states, and maintains compositional order against the constant pressure of microbial growth and environmental perturbation? We propose that this force is immune surveillance, and that its operation during adulthood reflects active, ongoing control, rather than passive tolerance of a resident community (Fig 1).

The concept of immune surveillance has been fruitful in cancer biology, where it refers to the continuous scanning of host tissues by innate and adaptive immune cells, eliminating transformed or aberrant cells before they can establish a tumor [26]. The underlying logic is one of ongoing active constraints: the immune system does not wait for disease to manifest but suppresses it continuously, as a baseline function of tissue maintenance. Tumor immunoevasion, the progressive acquisition by cancer cells of mechanisms that render them invisible or resistant to immune attack, is what happens when this constraint fails [27,28]. We would argue that an equivalent logic governs the host–microbiome relationship, with one important conceptual modification. In the tumor context, surveillance operates on a detect-and-eliminate logic directed at aberrant identity: transformed self is recognized and destroyed. Applied without modification to commensals, this logic would predict a sterile gut, which is clearly not the case. The key conceptual shift we propose is that immune surveillance of the microbiome is directed not at identity but at activity. Beyond damage-associated signaling (e.g., through damage-associated molecular patterns [29]), the host immune system does not primarily ask whether a given microorganism belongs to a recognized commensal taxon or is a known pathogen. It asks, continuously and across the full surface area of mucosal tissue, which organisms are growing, dividing, and expanding right now.

The molecular basis for this activity-sensing logic is already present in the known biology of innate immune recognition, though the signals involved reflect a mixture of two distinct inputs that the host must integrate: microbial load and microbial proliferation. These are related but not equivalent. A dense community of quiescent organisms presents a different immunological challenge than a sparse but rapidly dividing one, and the immune system appears to have distinct sensors for each.

Several canonical pattern recognition signals scale primarily with microbial biomass rather than growth rate, and are better understood as load sensors. Lipopolysaccharide, recognized by Toll-like receptor 4 (TLR4), and lipoteichoic acid, recognized by TLR2, are cell wall components that are shed in proportion to the quantity of bacteria present, whether dividing or not [30,31]. These receptors provide the immune system with a continuous readout of how much microbial material is present in a given compartment, which is necessary but insufficient for the homeostatic rule we propose. A second class of signals scales more specifically with proliferative activity and metabolic state, and these are the ones most directly relevant to the surveillance framework we develop here.

Peptidoglycan fragments detected by NOD-like receptors are generated during active cell wall remodeling throughout bacterial growth, making them a readout of metabolic and proliferative activity rather than biomass alone [32]. Flagellin, the structural subunit of bacterial flagella sensed by TLR5, is produced by motile bacteria actively navigating the mucosal environment; motility correlates with active growth, placing flagellin sensing somewhere between a load signal and a proliferation signal [33]. A third class of signals provides a readout that is orthogonal to both load and proliferative rate: TLR8, which detects single-stranded RNA, extends this surveillance to bacteria and to archaea, and because extracellular RNA is rapidly degraded by environmental RNases, steady-state TLR8 stimulation is expected to reflect living rather than dead microbial material, partially decoupled from cell wall-derived signals [11,34]. Whether it further discriminates metabolically active from quiescent cells at the molecular level remains unclear.

Burden, proliferative activity, and viability are non-redundant axes of microbial state, each requiring a dedicated sensing channel. How the host integrates these overlapping but distinct streams of information, tracking load, proliferative dynamics, and viability through partially separable sensing channels, into a unified homeostatic response remains incompletely understood, and represents one of the central open questions this framework raises. What is clear is that immunological visibility does not scale uniformly with microbial presence: a bacterium growing rapidly near a mucosal surface generates a qualitatively different signal profile than a quiescent one at the same abundance, and the immune response calibrated to that profile is correspondingly different. We propose that it is this proliferation-biased component of immune sensing, rather than load sensing alone, that constitutes the core homeostatic rule of microbiome maintenance during adulthood.

This activity-sensing logic is implemented at the cellular level by a layered architecture that distributes the receptors above (and additional sensors) across distinct effector populations. At the first line stand the epithelial cells themselves, equipped with pattern-recognition receptors that continuously sample luminal contents in the gut and calibrate the secretion of antimicrobial peptides and cytokines in response [35]. Behind this, the lamina propria harbors populations of innate lymphoid cells (ILCs 1–3), that integrate epithelial signals and shape the local cytokine environment, licensing or suppressing downstream immune responses depending on context [36]. ILCs also signal back to the epithelium, with ILC3-derived IL-22 in particular driving epithelial production of antimicrobial peptides (such as RegIIIγ) and defensins, thereby directly shaping the chemical landscape that constrains microbial growth at the mucosal surface [37]. Mucosal-associated invariant T cells (MAIT cells) represent a convergence of innate speed and adaptive specificity: they respond to riboflavin precursor metabolites produced by a broad range of bacteria, mounting rapid cytokine responses in a manner that is both taxonomically broad and tuned to metabolic activity [38]. Together, these populations constitute a surveillance network, the collective output of which is not wholesale elimination but continuous, graded calibration of microbial proliferative space.

Layered atop this innate sensing, mucosal surfaces are continuously coated with secretory antibodies at the adaptive layer, predominantly IgA but also IgM and, in some compartments, IgD, the precise targets of which within the microbial community and the functional consequences for individual taxa remain an active and genuinely unresolved area of investigation. Secretory IgM, far from being a mere compensatory isotype in IgA-deficient individuals, emerges from plasma cells clonally related to gut memory B cells and targets a highly diverse range of commensals under normal conditions [39], suggesting that mucosal antibody surveillance of the microbiome is broader in isotype scope than the IgA-centric literature has historically implied. IgA-sequencing approaches have produced complex and context-dependent findings regarding whether highly coated bacteria are preferentially commensals or pathobionts [40,41]. IgA preferentially binds immune-activating taxa, with the downstream outcome shifting from regulatory in health [42] to pathogenic in inflammatory contexts, where IgG further amplifies effector responses. Coating status alone is therefore insufficient to predict the functional consequence, which depends on antibody affinity, epitope specificity, the local Fc receptor landscape, and host inflammatory tone.

This complexity cautions against treating mucosal antibody responses as a simple targeting system with a fixed logic. What is clear, however, is that at least one mechanism operating within this antibody layer is intrinsically keyed to proliferative activity rather than to taxonomic identity. Antibody-mediated physical cross-linking of daughter cells at the moment of bacterial division, a process termed enchained growth, prevents their dispersal and limits colonization of new mucosal niches [43]. This mechanism does not require the immune system to distinguish commensal from pathobiont, nor does it depend on the downstream functional outcome of Fc receptor engagement: a quiescent bacterium produces no daughter cells and escapes enchainment entirely, while a rapidly dividing one is constrained precisely as it attempts to expand. Whether IgM and IgD contribute analogous proliferation-constraining mechanisms at mucosal surfaces, and how the relative contributions of different antibody isotypes to microbiome homeostasis change across the life span, remain open questions with direct relevance to understanding immune decline in aging.

**Fig 2:**
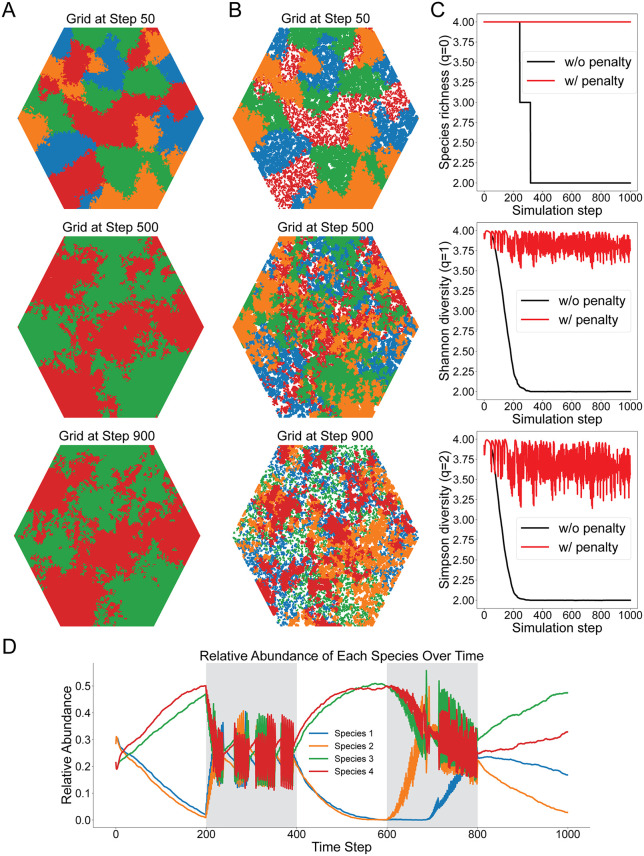
Immune surveillance decline enables microbial escape dynamics. A conceptual ecological/evolutionary model shows how reduced immune constraint permits microbial expansion and diversification. Four microbial species compete for space on a hexagonal lattice, with distinct replication outputs, simulated over 1,000 time steps. When abundance thresholds are exceeded (70% total abundance and 30% dominance), immune penalization removes 80% of cells. **A**. Baseline dynamics without immune penalization. **B**. Simulation with immune penalization applied throughout the entire simulation. **C**. Temporal dynamics of community diversity quantified by Hill numbers, including species richness (*q* = 0), Shannon diversity (*q* = 1), and inverse Simpson diversity (*q* = 2). **D**. Simulation with temporally intermittent immune penalization applied during steps 200–400 and 600–800. The prototype implementation of the model is available at: https://github.com/sql647/hexagonal_model.

What community-level outcome does this simple rule produce? We propose that a simple, two-threshold immune suppression rule, one that is triggered by overall microbial load and directed at whichever taxon has reached disproportionate relative abundance, is sufficient to maintain high taxonomic diversity as an emergent property, without requiring the host to actively recognize or curate individual community members (Fig 2). In our model, dominance is the proximate trigger for immune constraint, and dominance is itself the product of proliferative success: the taxon that grows fastest under current conditions is the one most likely to exceed the relative abundance threshold and attract suppression. Proliferative activity and dominance are therefore coupled in the model, as they likely are in vivo, even if the molecular sensors read them through partially distinct signals. When the suppression rule is removed, one or two species reach competitive dominance and diversity collapses, as measured across three representative diversity metrics (Hill diversity orders), ranging from simple species counts to measures increasingly weighted toward the most abundant taxa. When the two-threshold rule is active, high diversity is maintained as an emergent and stable outcome, and this result is robust across species with substantially different intrinsic proliferation rates, indicating that the mechanism does not depend on fine-tuned ecological parameters. The logic is structurally analogous to the ‘keystone predator’ concept in community ecology [44]: by preferentially targeting the most abundant taxon, the predator prevents any single competitor from monopolizing resources, and high diversity persists as the emergent consequence. In microbial ecology, a closely related principle has been formalized as the “kill-the-winner” hypothesis, originally developed for phage–bacteria dynamics in marine systems, where virus-mediated lysis of the most abundant bacterial strain prevents dominance and sustains community diversity [45]. We suggest that immune surveillance operates by an analogous logic at mucosal surfaces: by continuously suppressing whichever taxon has reached disproportionate abundance, and thereby constraining the proliferative expansion that produced that dominance, the immune system prevents any single strain from achieving the effective population size necessary to dominate the community metabolic space.

This concept reframes microbiome diversity not as a fixed property of a healthy host but as a dynamic equilibrium emerging from ongoing immune activity. It also generates a non-obvious prediction with direct relevance to aging: the collapse of microbiome diversity observed in old age need not reflect a specific failure to protect any particular commensal taxon. It may instead reflect the generalized erosion of the dominance-sensing constraint that had been preventing competitive exclusion all along. When immune pressure relaxes, the most proliferatively competitive taxa, which are often those with the broadest metabolic flexibility and the greatest capacity for opportunistic pathogenesis, expand into the space that surveillance had been holding open for the community as a whole.

The host, however, may pay a cost for maintaining this ecological state. A diverse microbiome sustained by active immune constraint will typically include organisms capable of harm under permissive conditions. Pathobionts, defined as commensal taxa with pathogenic potential that is expressed only when host defenses are compromised, are a predictable feature of any system in which immune surveillance enforces coexistence rather than selective elimination [46]. The presence of low-level *Clostridioides difficile* in the gut of a substantial fraction of healthy adults illustrates this logic directly: the organism is held in check not merely by immune activity but by the competitive pressure of the diverse community that immune surveillance maintains around it. Antibiotic treatment collapses that ecological layer of protection; immune compromise removes the surveillance layer. When both fail simultaneously, as they often do in older hospitalized patients, *C. difficile* expansion follows with clinical reliability [47]. Diversity is not, in this framework, an unconditional good. It is a conditionally stable state that depends on the immune system’s continued capacity to enforce the rule that created it. The benefits are real, if not always fully quantified: a diverse microbial consortium provides the host with access to a broader metabolic space than any single dominant taxon could, covering vitamin synthesis, bile acid transformation, and short-chain fatty acid production across a wider range of substrates and conditions. A further, more speculative benefit operates at the evolutionary timescale: by preventing any taxon from reaching the large population sizes required for rapid adaptive evolution, immune-maintained coexistence may reduce the probability that pathogenic traits accumulate within the community over time. Whether and how hosts calibrate this tradeoff across the life span, balancing these benefits against the risks of harboring potential pathobionts, remains an open and largely unaddressed question.

A minimal formalization helps to make the logic explicit. Consider a community of *n* microbial taxa, each with an intrinsic growth rate modified by competitive interactions and by an immune suppression term that is triggered when total community abundance exceeds a load threshold and when any single taxon exceeds a relative abundance threshold. Any taxon approaching dominance experiences disproportionate immune constraint, stabilizing the system around a state of high diversity. Remove or reduce the immune suppression term, and the system converges toward competitive exclusion, with the fastest-growing taxon displacing others. This is not a quantitative model with fitted parameters; it is a conceptual skeleton, intended to show that the proposed rule is logically sufficient to produce the observed community-level outcome. Fig 2 demonstrates this formally: in simulations without immune suppression, diversity collapses as one or two taxa reach competitive dominance (Fig 2A); when the two-threshold suppression rule is active, all three diversity indices are maintained at high values throughout the simulation, and this result holds across species with different intrinsic proliferation rates (Fig 2B and 2C).

Intrinsic ecological forces, including resource competition, spatial exclusion along mucosal crypts, pH and oxygen gradients, and dietary substrate availability that itself shifts with age, contribute to community stability and cannot be dismissed. These forces also set an absolute upper bound on community size through resource limitation and carrying capacity. But they are insufficient on their own. Loss of adaptive humoral immunity is associated with microbiome disorganization in humans, as demonstrated by altered community composition and reduced diversity in individuals with selective IgA deficiency [48]. The ecological scaffolding alone does not hold. What immune surveillance provides is the extrinsic force that imposes and maintains compositional order on an otherwise open and dynamic ecological system. How the host calibrates its sensing of microbial load versus community composition, how surveillance thresholds are set and adjusted across mucosal compartments, and whether these thresholds shift with age in ways that precede or merely accompany the compositional changes associated with aging, are questions that remain unresolved and that longitudinal immune–microbiome co-profiling studies are only beginning to address.

## How is microbial stability maintained in other ecosystems?

The host–microbiome surveillance framework developed above gains explanatory power when placed in comparative context. Microbial communities associated with plants and soil ecosystems display stability properties structurally analogous to those described for animal gut microbiomes, and the mechanisms sustaining that stability reveal a consistent logic: community order emerges from the superposition of intrinsic ecological constraints and extrinsic immune-like suppression, neither of which is sufficient alone.

Soil microbial communities are among the most diverse on Earth, and their compositional stability across disturbance events has long been attributed primarily to the physical and chemical complexity of the soil environment, which creates a vast number of distinct microhabitats that different organisms can occupy without directly competing [49]. Yet bacteriophage communities in soil impose kill-the-winner dynamics on the most abundant bacterial taxa, providing an extrinsic suppression mechanism that operates by a logic directly parallel to that proposed here for the specific sensors in mucosal immune surveillance [50]. The rhizosphere, the chemically distinct zone of soil shaped by root secretions, hosts microbial communities that are actively sculpted by the plant itself. Root exudates actively sculpt rhizosphere community composition by selectively enriching specific taxa, a form of chemical niche construction directly analogous to the selective enrichment of gut commensals by host-produced prebiotics [51]. Plant innate immune recognition at the root surface shapes microbial community composition independently of root exudate chemistry. When this recognition is genetically disrupted, the rhizosphere community is altered and fails to support normal nutrient stress responses [52], demonstrating that immune control and chemical niche construction operate as complementary rather than redundant layers. The leaf microbiome adds a further dimension. Loss of specific hub fungal taxa, including Oomycetes, produces bacterial blooms that do not occur when the full community is intact [53]. Natural genetic variation in immune genes across wild *Arabidopsis* populations directly shapes the bacterial communities on leaf surfaces, independently of local environmental conditions, establishing host immune genotype as a direct determinant of leaf microbial community composition [54]. The convergence of evidence across soil, root, and leaf systems supports a general principle: intrinsic ecological forces set the boundaries of possible community states, but extrinsic immune-like suppression is required to maintain compositional fidelity within those boundaries, and the relative weight of each layer scales with the degree to which the host has invested in active microbial management.

That the same two-layer logic recurs across plants, soil, and animal systems strengthens the inference that human age-associated dysbiosis reflects the breakdown of an active surveillance layer that has been holding the community together throughout adulthood, rather than passive ecological drift uncovered by other aging changes.

## What changes with age?

The gut microbiome undergoes consistent and reproducible changes during aging across species. Centenarians, whose microbiomes are characterized by depletion of opportunistic taxa and enrichment of health-associated lineages [55,56], illustrate that what healthy aging preserves is resistance to pathobiont expansion and the maintenance of health-associated community states, rather than any particular diversity target. What late-life dysbiosis disrupts is precisely this stability: across multiple independent human cohorts, aging is associated with declining evenness, loss of core taxa, bloom of opportunistic lineages, and increased inter-individual divergence, a pattern linked to metabolic dysfunction, frailty, and mortality [57]. Similar trajectories have been documented in other mammals and in the African turquoise killifish, where microbiome deterioration precedes and predicts organismal decline [15]. The consistency of this pattern across phylogenetically distant taxa suggests a conserved mechanism of failure.

We propose that mechanism is immunosenescence. The aging immune system undergoes pervasive functional remodeling across multiple axes simultaneously. Innate immune cells lose responsiveness to novel stimuli while becoming increasingly prone to constitutive, low-grade activation; the result is ‘inflammaging’: a chronic inflammatory tone driven by NF-κB activation in senescent stromal and immune cells, increased circulating pro-inflammatory cytokines such as IL-6, IL-1β, and TNF, and continuous innate stimulation by microbial products translocating across a progressively leaky epithelial barrier [58]. Adaptive immunity also deteriorates through distinct mechanisms. Naive T cell output declines as thymic involution and hematopoietic stem cell aging reduce lymphoid progenitor production [59,60]. The regenerative capacity of resident mucosal immune populations declines, as many of these populations, including tissue-resident ILCs, are maintained primarily by local self-renewal rather than continuous central replenishment [61]. MAIT cell numbers also decline substantially with age in peripheral blood [62], reducing the pool available for mucosal surveillance. While total mucosal and circulating IgA frequently increases with age, this increase is dominated by polyreactive, T-cell-independent, low-affinity IgA, reflecting chronic stimulation and barrier compromise, rather than expansion of antigen-specific responses [63]. The high-affinity, germinal-center-derived IgA repertoire that mediates discriminating microbial recognition declines with age through reduced germinal center activity at mucosal sites, impaired T follicular helper cell function, B cell senescence, and altered plasma cell trafficking, with likely consequences for microbial community composition [64–66]. The diversity of antigen-specific responses erodes unevenly with age, as clonal expansions driven by persistent pathogens progressively dominate the memory compartment at the expense of naive precursor breadth [67]. The net effect is not simply a weaker immune system but a reorganized one, trading precision and responsiveness for a constitutive, poorly discriminating background of inflammation.

In the context of the surveillance framework developed above, this reorganization maps onto a specific failure mode. The load-sensing arm of mucosal immunity, driven by TLR2 and TLR4 recognition of bacterial cell wall components, may remain constitutively triggered in the aging gut, perpetually signaling high microbial biomass, while the proliferation-discriminating functions that enforce the dominance-suppression rule progressively erode. This asymmetry is mechanistically plausible: constitutive pattern-recognition receptor signaling on innate effector cells persists independently of lymphocyte renewal, germinal center function, and receptor diversity, capacities that erode most prominently with age. The first threshold of the surveillance model is permanently breached; the second goes unenforced. Age-associated dysbiosis is therefore not simply the consequence of absent immune pressure, but of misdirected immune activity replacing precise surveillance. Whether this failure mode is empirically borne out in the aging gut remains to be directly tested.

Natural experiments support the causal direction, and partially recapitulate the failure mode. HIV infection (which preferentially depletes CD4^+^ T cells from gut-associated lymphoid tissue while simultaneously driving chronic immune activation and systemic inflammation) produces dysbiosis, the severity of which tracks nadir CD4^+^ T cell counts independently of behavioral confounders [68], modeling in accelerated form both the loss of mucosal surveillance capacity and the inflamed immune background that characterize aging. Selective IgA deficiency in humans is associated with direct shifts in microbiome composition and reduced diversity [49], establishing immune status as an operative causal variable in community structure. What follows from relaxed immune constraints is not random. The result is a directed compositional shift: a bloom of opportunistic taxa at the expense of core commensals, the ecological signature of which matches what aging cohort data consistently show [57,69].

Whether this represents purely ecological restructuring or also involves within-host microbial evolution remains an open question. Ecological escape operates on the timescale of community turnover, days to weeks; evolutionary escape, the accumulation of immune-evasion traits within expanding lineages, operates more slowly but would be expected to produce changes that are harder to reverse. Both are plausible across the timescale of human aging [70], and distinguishing them requires longitudinal metagenomic approaches with sufficient resolution to track within-strain variation alongside community dynamics [71].

## Can immune function stabilize the aging microbiome?

The surveillance framework developed here carries a direct therapeutic implication that inverts the logic of most current microbiome-targeted interventions. If age-associated dysbiosis is a downstream consequence of immune decline rather than a primary event, then restoring immune surveillance capacity is a more causally targeted intervention than manipulating community composition directly. Several immune-restorative strategies are biologically motivated in this context, although evidence linking them specifically to microbiome stabilization remains limited.

IL-7 administration expands naive T-cell compartments and increases T-cell receptor repertoire diversity in lymphopenic humans, primarily through preferential expansion of naive T-cell subsets [72], addressing one axis of adaptive immune erosion. Partial thymic regeneration has been reported in older humans following a combined hormonal intervention [73], although microbiome outcomes were not assessed. Senolytic clearance of senescent cells reduces the constitutive NF-κB-driven inflammatory tone that, in the surveillance framework, represents the permanent breach of the load-sensing threshold without corresponding enforcement of the dominance-suppression rule, generating inflammatory noise where precise community-level constraint should operate [74]. Whether any of these interventions is sufficient to restore the proliferation-discriminating functions of mucosal immunity, rather than merely reducing background inflammation, is not known and represents a direct experimental priority that follows from this framework.

The caution runs in the opposite direction for microbiome-targeted interventions applied without regard for immune context. Probiotics carry documented risks of bacteremia and fungemia in immunocompromised individuals [75], and fecal microbiota transplantation has caused transmission of drug-resistant organisms and death in recipients with compromised immune function [76]. The surveillance framework provides a mechanistic rationale for these observations: introducing diverse microbial taxa into a host who has lost the capacity to constrain them removes the ecological benefit of diversity while retaining its risks. In the most severely immunocompromised clinical contexts, gut decontamination rather than microbial enrichment has historically been practiced in some transplant settings [77], although its benefits remain debated, and this clinical intuition is broadly consistent with the logic developed here.

**Fig 3:**
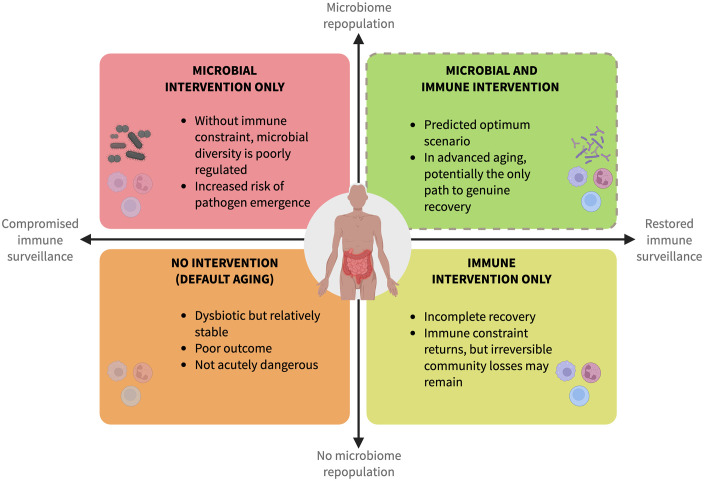
Intervention space for age-related gut microbiome dysbiosis. The intervention space is organized along two axes: immune surveillance capacity (compromised to restored) and microbiome repopulation (absent to active). The framework addresses trajectories in which selective core taxa have been irreversibly lost, a subset of aging outcomes shaped by phenotypic heterogeneity among older individuals. Each quadrant represents a distinct combination of immune and ecological interventions, with predicted outcomes ranging from poor (no intervention, default aging) to optimal (combined immune restoration and ecologically informed repopulation). The dashed border of the upper-right quadrant indicates that this combined strategy is predicted but not yet empirically demonstrated. Figure created in BioRender. *Costa, F. (2026)* https://BioRender.com/soo16vu.

Durable microbiome stabilization in aging is unlikely to be achieved by ecological management alone. Immune restoration is best understood as the prerequisite that makes microbiome restoration therapeutically productive, particularly for re-seeding irreversibly lost taxa that immune intervention alone cannot recover. The most productive interventional strategy is probably one that addresses immune surveillance capacity and community composition simultaneously, treating them as coupled rather than independent targets (Fig 3).

## Toward a unified theory of immune–microbiome aging

The framework developed here reframes a familiar observation, that aging disrupts the gut microbiome, as the downstream consequence of a specific and in principle modifiable failure: the progressive erosion of immune surveillance capacity that had been actively constructing and maintaining the microbial niche throughout adulthood. Several questions follow directly from this reframing and remain unresolved.

The most pressing is mechanistic: which components of mucosal immune surveillance are rate-limiting for community stability? The framework implicates the proliferation-discriminating functions of innate and adaptive immunity over load-sensing, but the relative contributions of specific cell populations (including MAIT cells, ILC3s, mucosal IgA^+^ plasma cells, and the epithelial pattern recognition layer) to the dominance-suppression rule have not been dissected in vivo. Whether any single axis of immune decline is sufficient to initiate dysbiosis, or whether community collapse requires the simultaneous erosion of multiple layers, is not known. Conditional ablation of specific immune compartments in defined mucosal niches, combined with longitudinal metagenomic profiling, offers a direct experimental path. The framework predicts that loss of proliferation-discriminating components should produce dominance shifts and opportunist expansion, whereas loss of load-sensing components alone should not.

The timescale question is equally open. Ecological escape and evolutionary escape operate on different timescales and produce qualitatively different kinds of dysbiosis, the first reversible in principle, the second increasingly resistant to correction as immune-evasion traits accumulate within expanding lineages. Distinguishing these two processes will require longitudinal metagenomic approaches with sufficient resolution to track within-strain variation alongside community dynamics, a technical capability that is only now becoming tractable in human cohorts [78]. Whether windows of intervention exist, periods in the aging trajectory where immune–microbiome coupling remains intact enough that restoring surveillance capacity would reverse rather than merely slow community deterioration, remains entirely unaddressed.

The most tractable experimental entry points are probably in short-lived model organisms with defined microbiomes. The African turquoise killifish, the microbiome deterioration of which precedes and predicts organismal decline, has an immune system that is accessible to genetic manipulation and offers a particularly direct path to testing whether immune reconstitution stabilizes the microbiome in vivo. Drosophila and zebrafish offer complementary entry points for dissecting innate and adaptive immune contributions separately: *Drosophila* lacks adaptive immunity entirely, making it suited to isolating the innate surveillance arm, whereas zebrafish combine a well-characterized adaptive immune system with genetic tools that allow cell-type-specific manipulation. Together, these systems provide a tractable experimental ladder for dissecting which layers of immune surveillance are rate-limiting for microbiome stability.

The holobiont framework, which conceptualizes host and associated microbiome as a single unit of selection in evolution, has recently emphasized the cooperative and mutually beneficial dimensions of host–microbiome relationships [79]. The dynamics described here suggest a more unsettled picture. The host–microbiome relationship is better understood as an agora, a dynamic forum of cooperation, competition, and exploitation, the stability of which depends to a large extent on the host’s continued capacity to set and enforce the rules of engagement. When that capacity erodes, the most opportunistic community members expand into the space that surveillance has maintained. Immune-centered microbiome research, treating surveillance not as a background condition but as the active organizing principle of microbial ecology across the life span, represents a reorientation with direct implications for how aging biology and microbiome science are integrated going forward.

## Abbreviations

ILCs: innate lymphoid cells
MAIT: mucosal-associated invariant T
TLR4: toll-like receptor 4
